# An infant with anaphylaxis

**DOI:** 10.1186/2045-7022-1-S1-S16

**Published:** 2011-08-12

**Authors:** Graham Roberts

**Affiliations:** 1 Southampton University Hospital NHS Trust, Paediatric Allergy and Respiratory Medicine, University Child Health, Southampton, UK

## 

A 4 month old infant presents to your hospital with a history of becoming unwell with difficulty in breathing after being given a different cows milk formula. She had previous devevloped urticaria with some porriage.

This case incorporates a few common clinical challenges: how to recognise anaphylaxis in infants, identifying the responsible allergen and determining the most appropriate anaphylaxis management plan.

Recognition of anaphylaxis in infancy: anaphylaxis can be difficult to recognise in infants and readily confused with other presentations (Simons 2007).

Finding the responsible allergen: in this case, there is seemingly no contact with a new food. The challenge is that food manufacturers use multiple ingrediates in processed food so that a cows milk formula will not only include cows milk (Roberts 2008). Additionally other non food allergens should be considered, examples are latex and insect stings. Some food may only behave as allergens with a co-factor such as exercise. Skin prick testing and specific IgE may be helpful in clarifying the allergen although care is required when interpreting results with a recent anaphylactic reaction or in a young infant (Roberts 2007). Lastly, there are other conditions that have a similar presentation to anaphylaxis (Muraro 2007).

Prescribing an appropriate anaphylaxis management plan: the smallest available autoinjector contains 0.15mg adrenaline and is licenced only from 15kg. But we know that parents take a longtime to draw up adrenaline from ampoule and are unable to accurately draw up the correct volume. Furthermore we know anecdotely that when parents try to treat reactions themselves, they delay calling the emergency services. We therefore have a dilema as to what is the best anaphylaxis management plan for a small infant: an addrenaline autoinjector which will deliver an overdose, a syringe and ampoule of adrenaline which we know parents find difficult to use or a plan that asks parent to call very early for the emergency services. Difference plans may be most appropriate for different infants and the best plan may change as they grow.

Finally a young infant with food allergy needs regular follow to provide parents with advise about their diet, monitor the infant’s growth, alter the anaphylaxis management plan as required and retesting to determine whether or not the infant the has outgrown their food allergy.
